# Post-stroke dysphagia across the care continuum: a full-cycle framework for rehabilitation, supportive care, and community follow-up

**DOI:** 10.3389/fneur.2026.1886974

**Published:** 2026-09-03

**Authors:** Jiayi Xia, Song Pei

**Affiliations:** 1 Clinical Research Center, Shanghai Second Rehabilitation Hospital, Shanghai University of Traditional Chinese Medicine, Shanghai, China; 2 Department of Rehabilitation Medicine, Renhe Hospital, Baoshan District, Shanghai, China

**Keywords:** aspiration pneumonia, community follow-up, continuity of care, deglutition disorders, post-stroke dysphagia, stroke rehabilitation, swallowing rehabilitation, transitional care

## Abstract

Post-stroke dysphagia is a common and clinically consequential complication of stroke, associated with aspiration pneumonia, malnutrition, dehydration, impaired functional recovery, mortality, and increased healthcare use. Although acute dysphagia screening and aspiration-risk management are increasingly embedded in organised stroke care, long-term reassessment, discharge transition, caregiver education, community follow-up, and digitally supported continuity of care remain less clearly defined. This Review synthesises current evidence on post-stroke dysphagia management within a full-cycle clinical framework extending from hyperacute screening to long-term community care. High-consensus practices include standardised screening before oral intake, timely escalation to clinical swallowing examination and instrumental assessment in high-risk patients, and early supportive decisions regarding feeding route, hydration, medication administration, positioning, oral hygiene, nutrition support, and pulmonary surveillance. Standard swallowing rehabilitation remains the platform from acute rehabilitation through the subacute phase; targeted exercise, electrical or brain stimulation, respiratory muscle training, and biofeedback are optional phenotype-based intensification modules, not universal treatments. Beyond the inpatient episode, care pathways should specify swallowing status, aspiration risk, diet and fluid recommendations, feeding route, oral-care instructions, warning signs, reassessment timing, responsible services, caregiver support, and escalation rules. Palliative care supports preference-sensitive prognosis, symptom, or feeding decisions; community implementation must address access, caregiver capacity, and loss to follow-up. Future research should move beyond isolated short-term swallowing outcomes and evaluate integrated hospital-home-community models using clinically meaningful endpoints, including pneumonia, nutritional recovery, readmission, mortality, patient-reported outcomes, social participation, and caregiver burden. Managing post-stroke dysphagia as a cross-stage, cross-setting, and multidisciplinary stroke-care problem may improve safety, continuity, and long-term recovery.

## 1 Introduction

### 1.1 Clinical importance of PSD

Post-stroke dysphagia (PSD) is one of the most common and clinically consequential complications after stroke. Contemporary evidence suggests that dysphagia affects roughly 40%–50% of patients after acute stroke, although reported estimates vary depending on case mix, timing of assessment, and diagnostic method (1, 2). PSD is not merely a problem of oral intake. It is strongly associated with aspiration pneumonia, dehydration, malnutrition, longer hospitalisation, poorer functional recovery, increased mortality, and higher healthcare expenditure (1–3). In the European Stroke Organisation and European Society for Swallowing Disorders (ESO/ESSD) guideline, PSD is explicitly framed as a complication that requires systematic screening, assessment, and treatment because of its close link to post-stroke pneumonia, early mortality, and poor outcome (1).

The clinical importance of PSD also extends beyond the acute admission. Even when some patients resume oral feeding, swallowing impairment may persist as dietary restriction, fear of eating, reduced social participation, and impaired quality of life, indicating that PSD should not be viewed solely as a transient inpatient safety issue (1). For clinicians, this means that PSD is best understood not as an isolated symptom, but as a multidimensional stroke-related disorder with prognostic, nutritional, respiratory, functional, and economic consequences across the continuum of care (1–3).

### 1.2 Why a full-cycle perspective is needed

A full-cycle perspective is needed because the current management of post-stroke dysphagia remains structurally unbalanced. In most stroke services, the acute phase is comparatively well defined: early dysphagia screening, aspiration-risk identification, and escalation to instrumental assessment are increasingly embedded in organised stroke care pathways (1, 4). By contrast, what happens after the initial inpatient stage is far less clear. Recent analysis of international guidance has shown that recommendations for long-term reassessment, nutritional targets, oral care, caregiver education, and community follow-up remain sparse or inconsistent, leaving a substantial gap between early hospital-based management and chronic-phase care (5). As a result, the front end of PSD management is often protocolised, whereas the back end is fragmented.

This imbalance matters because PSD is not confined to the hyperacute admission. Stroke systems of care are increasingly expected to provide continuity across acute treatment, rehabilitation, discharge transition, and long-term recovery, yet dysphagia pathways have not evolved to the same extent (1, 4–6). Patients may leave hospital with broad advice on diet texture or swallowing exercises, but without a clearly defined reassessment interval, responsible follow-up team, or structured linkage to community support (5). Emerging real-world evidence further suggests that organised dysphagia management is associated with lower mortality after stroke, reinforcing the view that care quality across the pathway may itself influence outcome rather than merely reflect disease severity (7). A full-cycle framework is therefore needed to connect early safety management with subacute rehabilitation, supportive care, discharge planning, and long-term follow-up in a single clinically executable model (1, 4–7).

### 1.3 Aim of this review

The aim of this review is not simply to compare individual rehabilitation techniques for post-stroke dysphagia (PSD), but to synthesise current evidence within a clinically oriented full-cycle management framework. Specifically, this review examines how PSD should be managed across the continuum of care, from hyperacute screening and risk stratification to instrumental assessment, subacute rehabilitation, nutritional and oral-care support, discharge transition, and long-term community follow-up. By moving beyond a technique-centred perspective, we seek to address a more clinically relevant question: how dysphagia management can be organised across stages and settings to support safer care, more consistent decision-making, and better long-term outcomes after stroke.

**Figure 1:**
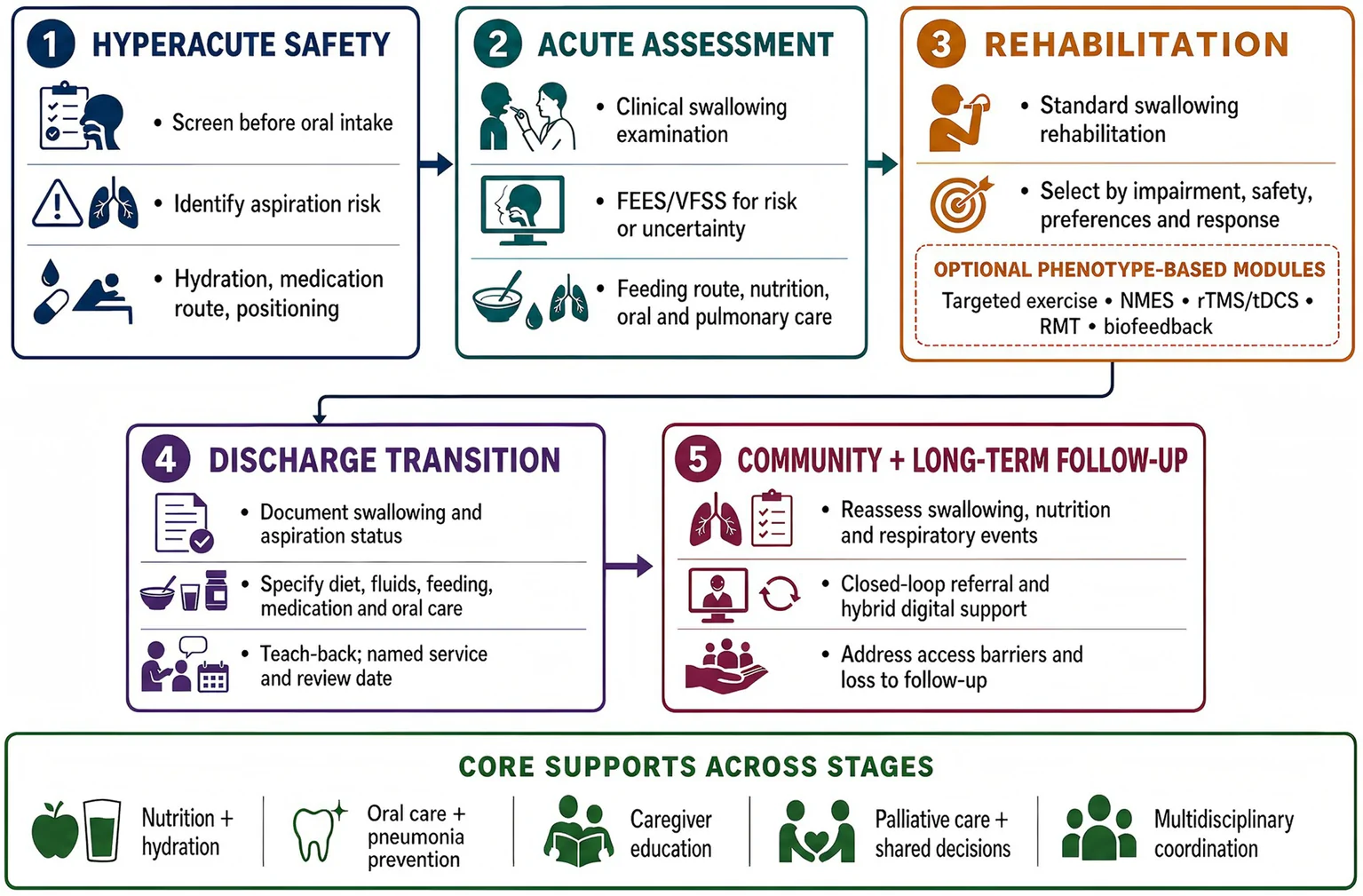
Full-cycle management framework for post-stroke dysphagia. The pathway links five clinically related stages: hyperacute safety, acute assessment, rehabilitation, discharge transition, and community and long-term follow-up. These stages represent changing care priorities and may overlap rather than fixed time windows; hyperacute screening and acute assessment may occur during the same admission, and rehabilitation may begin once medical stability and participation permit. Standard swallowing rehabilitation forms the therapeutic foundation, while targeted exercise, NMES, rTMS/tDCS, RMT, and biofeedback may be selected as optional phenotype-based modules according to the target impairment, safety, patient preferences, available resources, response, and tolerance. Core supports across stages include nutrition and hydration, oral care and pneumonia prevention, caregiver education, palliative care and shared decision-making, and multidisciplinary coordination. In this framework, pneumonia prevention denotes risk-reduction measures rather than guaranteed prevention, and palliative care and shared decision-making are applied when clinically indicated. FEES, flexible endoscopic evaluation of swallowing; NMES, neuromuscular electrical stimulation; RMT, respiratory muscle training; rTMS, repetitive transcranial magnetic stimulation; tDCS, transcranial direct current stimulation; VFSS, videofluoroscopic swallowing study.

This review therefore aims to provide a state-of-the-art, practice-relevant overview for clinicians involved in stroke medicine, rehabilitation, and post-acute care. In doing so, it highlights areas of high consensus, identifies domains in which evidence remains conditional or incomplete, and emphasises the need to connect acute safety management with longer-term continuity of care. The proposed full-cycle management framework is summarised in Figure 1.

Unlike technique-centred reviews that compare individual interventions, this Review contributes an implementation-oriented synthesis that links stage-specific clinical goals, supportive care, care transitions, accountability, and follow-up barriers within one hospital–home–community pathway. It does not claim new treatment-effect estimates; rather, it identifies where recommendations are guideline-supported, conditional, or primarily practice-based.

### 1.4 Search strategy and selection criteria

This narrative Review was conducted and reported with attention to the Scale for the Assessment of Narrative Review Articles (SANRA), while recognising that SANRA is an appraisal framework rather than a systematic-review reporting standard (8). The final search update was completed on 31 May 2026. We searched PubMed, Web of Science, Embase, the Cochrane Library, and Google Scholar for English-language literature concerning adults with dysphagia after either ischaemic or haemorrhagic stroke, from hyperacute care through long-term community follow-up.

Searches combined stroke terms (stroke, post-stroke, ischaemic stroke, haemorrhagic stroke) with dysphagia terms (dysphagia, deglutition disorder, swallowing) and topic blocks covering screening and assessment; FEES and VFSS; rehabilitation and therapy; nutrition, enteral feeding, oral care, and pneumonia; discharge and transitional care; community rehabilitation; caregiver experience and quality of life; telemedicine and digital health; and palliative care. Reference lists of key guidelines and reviews were searched for additional eligible sources.

Guidelines and consensus statements, systematic or scoping reviews, meta-analyses, randomised trials, cohort studies, qualitative studies, and key methodological papers were eligible when they informed management or organisation of post-stroke dysphagia care. We excluded paediatric or non-stroke dysphagia, reports without a management-relevant contribution, redundant or superseded publications, and non-English reports unless they supplied unique essential information. JX performed the search and initial eligibility review; SP independently verified eligibility of the final reference set, and differences were resolved by discussion.

**Figure 2:**
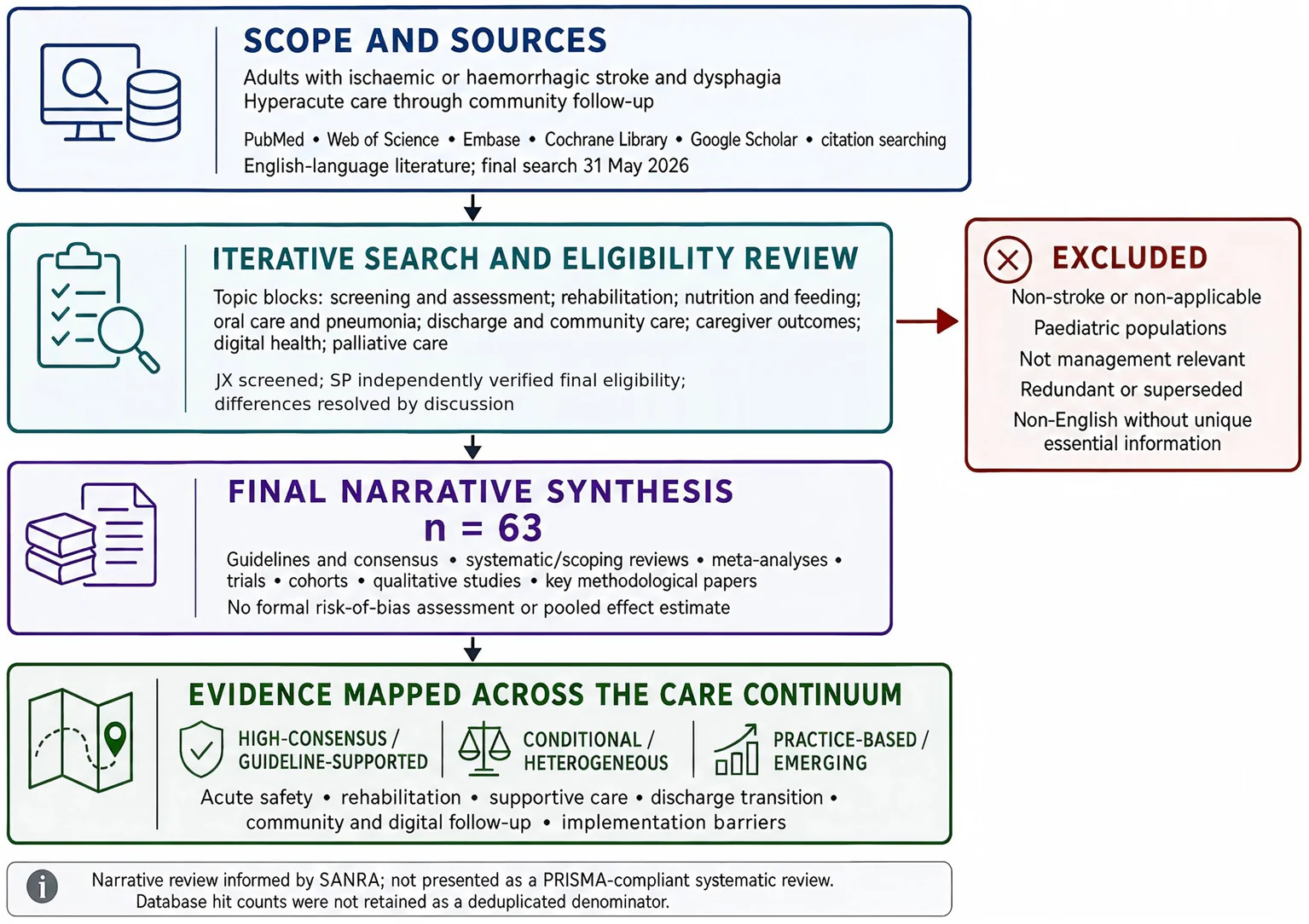
Narrative-review search and evidence-selection pathway. The primary clinical scope was adults with dysphagia after ischaemic or haemorrhagic stroke across the care continuum; directly applicable broader stroke-care sources were also used for implementation context. Searching was iterative across predefined topic blocks and supplemented by citation searching. JX screened the sources, SP independently verified the eligibility of the final reference set, and differences were resolved by discussion. The “*n* = 63” label denotes 63 cited sources—including guidelines, consensus statements, reviews, trials, cohort studies, qualitative studies, and methodological papers—rather than 63 primary studies. Raw database hit counts were not retained as a deduplicated systematic-review denominator; therefore, no post-hoc record-level exclusion counts are presented. The search focused on English-language literature; the non-English criterion shown in the figure represents an exception for unique essential information rather than routine inclusion of non-English reports. The evidence tiers are descriptive narrative categories and do not constitute formal consensus ratings, risk-of-bias assessments, or GRADE certainty ratings. SANRA, Scale for the Assessment of Narrative Review Articles.

The final narrative synthesis included 63 sources. Because the searches were iterative across topic blocks and Google Scholar rankings were dynamic, raw database hit counts were not retained as a deduplicated systematic-review denominator; consequently, a reliable *post-hoc* count of all unique records retrieved and excluded cannot be reported without creating false precision. Figure 2 documents the sources, eligibility logic, and final included set.

For interpretation, evidence was mapped into three narrative tiers: high-consensus or guideline-supported practice; conditional evidence characterised by heterogeneity or limited certainty; and practice-based or emerging proposals requiring prospective evaluation. These labels are used to separate established recommendations from expert interpretation, particularly for discharge planning, digital follow-up, community implementation, and palliative care; they are not a substitute for formal GRADE assessment.

The Review addresses multiple clinical questions, populations, interventions, settings, and outcomes across the care continuum. It was therefore not designed as a systematic review, and no formal risk-of-bias assessment or pooled quantitative synthesis was performed. Technique-specific pooled effects are represented through the cited systematic reviews and meta-analyses, while the present synthesis focuses on how evidence can be organised into an executable longitudinal care pathway.

## 2 Why post-stroke dysphagia requires a full-cycle management model

### 2.1 Burden beyond swallowing impairment

The burden of post-stroke dysphagia should not be judged solely by whether oral feeding is possible. Return to oral intake is important, but it does not capture the respiratory, nutritional, functional, psychosocial, and economic consequences of dysphagia after stroke. Meta-analytic evidence shows that dysphagia in acute stroke is associated with substantially increased risks of pneumonia and mortality (2). Cohort data also link dysphagia with dehydration, malnutrition, respiratory complications, and poorer recovery, particularly in older or medically vulnerable patients (9, 10).

Nutritional compromise is a key mechanism through which PSD may limit rehabilitation. Inadequate oral intake, prolonged texture modification, dehydration, or delayed optimisation of feeding route may reduce training tolerance, contribute to muscle loss, and increase infection risk (10, 11). Aspiration pneumonia is also not determined by bolus misdirection alone; oral microbial burden, impaired cough, reduced consciousness, immobility, comorbidity, and nursing quality all influence whether aspiration becomes pulmonary infection (1, 2). PSD should therefore be viewed as a syndrome in which airway protection, nutrition, oral health, respiratory reserve, and rehabilitation capacity interact.

The impact also extends beyond biomedical complications. Persistent dysphagia may cause fear of eating, dietary restriction, social withdrawal, reduced confidence, and impaired quality of life (12). It may also increase caregiver workload, especially when discharge instructions are vague or reassessment pathways are unavailable (12, 13). Dysphagia also adds measurable cost during the first year after stroke (3). Thus, PSD should be framed as a multidimensional determinant of respiratory safety, nutritional recovery, rehabilitation participation, caregiver burden, and healthcare utilisation.

### 2.2 Pathophysiological and clinical heterogeneity

Swallowing depends on integrated cortical, subcortical, cerebellar, brainstem, and peripheral sensorimotor pathways. Voluntary and reflexive swallowing require coordinated bilateral cortical activity, corticobulbar projections, basal ganglia and cerebellar circuits, brainstem swallowing pattern generation, cranial nerve nuclei, and peripheral sensory feedback (14–16). Post-stroke dysphagia reflects disruption of these complex neuroanatomical systems and their adaptive recovery mechanisms (17).

Lesion location matters, but does not act alone. A systematic review and meta-analysis found PSD-related structural or functional changes across multiple regions, including the sensorimotor cortex, insula, cerebellum, cingulate gyrus, thalamus, basal ganglia, and white matter connections (18). Clinical and imaging studies show that lesion location, stroke severity, and baseline clinical status influence both dysphagia occurrence and recovery after acute ischaemic stroke (15). MRI-based modelling also suggests that dysphagia, dysarthria, and aphasia after first acute ischaemic stroke have distinct but overlapping neuroanatomical predictors (16).

Brainstem lesions may directly affect the swallowing pattern generator and cranial nerve-mediated pharyngeal swallowing, whereas supratentorial lesions may impair swallowing initiation, sensory integration, voluntary control, and corticobulbar output (15–18). Lesion mapping further shows that different lesion locations may produce distinct physiological deficits in airway protection, bolus clearance, residue, or swallowing timing (19).

Clinical heterogeneity is equally important. Consciousness, attention, cognition, aphasia, neglect, frailty, respiratory reserve, cough strength, nutritional status, oral hygiene, and comorbidities may all modify aspiration risk and rehabilitation potential (1, 14, 20). Respiratory-swallow coordination is particularly relevant because safe swallowing requires precise timing between bolus transit, laryngeal closure, swallowing apnoea, and post-swallow expiration. Evidence from respiratory muscle training further supports the link between respiratory function, airway protection, and swallowing recovery (21).

Therefore, PSD should be understood as a heterogeneous syndrome rather than a uniform disorder. This heterogeneity supports a full-cycle model in which assessment, rehabilitation intensity, nutritional support, airway protection, and follow-up frequency are matched to patient phenotype, disease stage, and recovery trajectory.

### 2.3 Temporal shift in management priorities

The rationale for full-cycle management is that PSD priorities change over time. In the hyperacute phase, safety is dominant: dysphagia must be identified before oral intake or oral medication, and patients at aspiration risk should be triaged promptly. The ESO/ESSD guideline recommends formal dysphagia screening in all acute stroke patients and timely further assessment, including clinical swallowing examination and flexible endoscopic evaluation of swallowing (FEES) or videofluoroscopic swallowing study (VFSS) when screening is failed or clinical predictors are present. At this stage, management should focus on aspiration-risk identification, nil-by-mouth decisions when required, safe medication administration, hydration, early nutrition planning, and pulmonary surveillance (1, 11).

During acute and subacute rehabilitation, priorities shift toward functional recovery and complication prevention. Standard swallowing therapy, compensatory strategies, texture modification, nutritional support, oral care, and selected intensification modules should be integrated according to swallowing phenotype, stroke severity, tolerance, and rehabilitation goals (1, 22). The aim is to restore swallowing efficiency where possible, maintain nutrition and hydration, reduce respiratory complications, and support broader stroke rehabilitation.

After discharge, priorities shift again toward maintaining gains, adapting diet and exercises to real-world settings, reassessing swallowing status, and supporting caregivers. Intensified post-stroke care may improve dysphagia recovery within 1 year after acute ischaemic stroke (13). Qualitative evidence also shows that patients with PSD and caregivers face changing challenges across hospitalisation, rehabilitation transfer, and return home, underscoring the need for discharge planning, follow-up support, and community resources (12). A full-cycle model should therefore connect hyperacute safety management, subacute rehabilitation, and post-discharge reassessment into a continuous pathway supporting respiratory safety, nutritional recovery, social reintegration, and long-term stroke survivorship.

## 3 Hyperacute and acute care: screening, risk stratification, and instrumental assessment

### 3.1 Early screening before oral intake

Early dysphagia screening before oral intake is the first safety gate in hyperacute and acute stroke care. All patients with acute stroke should undergo standardised swallowing screening before receiving food, fluids, or oral medication, because undetected dysphagia may lead to aspiration, aspiration pneumonia, dehydration, malnutrition, and delayed rehabilitation (4). This recommendation is also consistent with stroke best-practice guidance, which explicitly recommends screening for swallowing impairment before any oral intake, including medications, food, or liquid (23).

The clinical value of early screening lies not only in identifying whether dysphagia is present, but also in preventing premature oral intake in patients with impaired consciousness, weak cough, poor secretion control, or silent aspiration risk. The ESO/ESSD guideline recommends formal dysphagia screening in all acute stroke patients and supports further dysphagia assessment when screening is failed or clinical predictors are present (1). Therefore, bedside screening should be embedded as a mandatory step within the acute stroke pathway rather than treated as an optional nursing task.

Screening should be performed by appropriately trained professionals using a validated tool and linked to predefined escalation rules. A positive or inconclusive screen should prompt temporary restriction of unsafe oral intake, appropriate hydration and nutrition planning, safe medication-route decisions, and timely referral for clinical swallowing examination or instrumental assessment when indicated (1, 11). In this sense, early screening is not the endpoint of diagnosis, but the entry point into a structured dysphagia management pathway.

### 3.2 Bedside screening as triage, not diagnosis

Bedside dysphagia screening should be understood as a triage procedure rather than a definitive diagnostic assessment. Its purpose is to rapidly identify patients who may be unsafe for oral intake and to determine the next step in the care pathway: supervised low-risk oral intake, temporary restriction of oral intake, clinical swallowing examination, or instrumental assessment (1, 4). This distinction is important because a screening test is designed for speed, feasibility, and risk detection, whereas diagnosis requires a more detailed assessment of swallowing physiology, airway protection, secretion management, residue, and response to compensatory strategies (1).

No bedside screening tool can fully replace formal swallowing assessment or instrumental evaluation. The ESO/ESSD guideline recommends formal dysphagia screening in all acute stroke patients but also recommends further dysphagia assessment when screening is failed or when clinical predictors of dysphagia are present (1). Recent evidence also suggests that screening tools vary in sensitivity, specificity, feasibility, and clinical applicability; a 2024 network meta-analysis found that Gugging Swallowing Screen (GUSS), Mann Assessment of Swallowing Ability (MASA), and Volume-Viscosity Swallow Test (V-VST) showed relatively favourable diagnostic performance, whereas simpler water-swallow approaches may require more selective use depending on patient condition and stroke stage (24).

Therefore, the clinical value of bedside screening depends less on identifying a single universally superior tool than on embedding the chosen tool within a clear escalation protocol. A failed or uncertain screen should trigger predefined actions, including continued restriction of unsafe oral intake, hydration and nutrition planning, safe medication-route decisions, referral to speech-language therapy or an appropriately trained dysphagia clinician, and consideration of FEES or VFSS when clinically indicated (1, 4, 11). In this sense, bedside screening functions as the front door to a structured PSD pathway, not as the endpoint of clinical decision-making.

### 3.3 When FEES or VFSS is required

Instrumental assessment should be considered when bedside screening fails, when the result is uncertain, or when clinical features suggest a high risk of aspiration despite an apparently acceptable bedside performance. According to the ESO/ESSD guideline, patients identified as being at risk of post-stroke dysphagia should undergo further dysphagia assessment, and instrumental methods such as FEES or VFSS are particularly relevant when clinical assessment alone cannot adequately define swallowing safety or physiology (1). In practice, high-risk situations include suspected silent aspiration, the overt aspiration warning signs defined in Section 3.4, poor secretion control, reduced consciousness, brainstem stroke, severe dysarthria or aphasia limiting bedside assessment, tracheostomy, prolonged nil-by-mouth status, or the need to make complex decisions about diet texture, feeding route, and rehabilitation strategy (1).

FEES and VFSS should be viewed as complementary rather than competing examinations. FEES can be performed at the bedside, repeated when needed, and is particularly useful for medically unstable patients because it directly visualises secretion management, pharyngeal residue, laryngeal penetration, aspiration before or after the swallow, sensory response, and airway protection (1, 25). It is therefore especially valuable when the main clinical question concerns secretion pooling, silent aspiration, laryngeal sensation, fatigue across repeated swallows, or whether oral intake can be safely initiated or advanced in the acute stroke setting (25).

VFSS provides a dynamic radiographic view of oral, pharyngeal, and upper oesophageal bolus transit and is particularly useful when clinicians need to analyse oral preparation, bolus propulsion, timing of swallow initiation, hyolaryngeal excursion, upper oesophageal sphincter opening, and the effects of different consistencies, postures, and compensatory manoeuvres (1, 26). Compared with FEES, VFSS offers a broader view of bolus flow across swallowing phases, but it requires patient transport, radiological resources, and radiation exposure, which may limit feasibility in medically fragile acute stroke patients (26).

The choice between FEES and VFSS should therefore be driven by the immediate clinical question, patient stability, local expertise, and availability rather than by a rigid hierarchy. FEES may be preferred when bedside accessibility, secretion assessment, repeatability, and aspiration-risk monitoring are central, whereas VFSS may be preferred when oral-phase biomechanics, bolus transit, upper oesophageal opening, or response to compensatory manoeuvres across consistencies must be clarified (1, 26). In both cases, instrumental assessment should not be treated as an isolated diagnostic event, but as a decision-support tool that informs diet prescription, feeding route, rehabilitation targets, aspiration-risk management, and reassessment planning.

### 3.4 Early supportive decisions

Once dysphagia risk is identified, acute management should move immediately from screening to supportive decision-making. Feeding route, hydration, medication administration, positioning, oral hygiene, and pulmonary surveillance should be treated as core components of PSD care rather than secondary bedside details. The ESO/ESSD guideline frames PSD as a multidisciplinary management problem because it increases the risk of aspiration pneumonia, malnutrition, dehydration, poor outcome, and mortality (1). Clinical nutrition guidance similarly emphasises that dysphagia, aspiration pneumonia, and insufficient nutritional intake contribute to worse outcomes after stroke, supporting early nutrition and fluid planning as part of acute stroke care (11).

Feeding route decisions should be linked to swallowing safety, expected recovery trajectory, nutritional risk, and patient tolerance. Patients with severe or prolonged dysphagia may require enteral feeding to maintain adequate nutrition and hydration while avoiding unsafe oral intake (11). Enteral nutrition should not be viewed as a failure of rehabilitation, but as a temporary or longer-term supportive strategy that preserves physiological reserve and enables participation in stroke recovery. Hydration requires equal attention, because inadequate fluid intake may occur silently in patients placed nil by mouth or maintained on poorly tolerated texture-modified diets (11, 27).

Medication route is another early safety decision. If swallowing is unsafe or uncertain, oral tablets or liquids should not be administered casually before dysphagia status is clarified. Instead, medication plans should be reviewed with the stroke team, pharmacy, nursing staff, and dysphagia clinicians to determine whether temporary non-oral routes, modified formulations, or supervised administration are required (1, 23). Positioning during feeding and after intake, monitoring for fatigue, and adapting meal pace or bolus size should also be integrated into early management because aspiration risk may fluctuate with arousal, posture, respiratory status, and endurance (1, 4).

Oral hygiene and pulmonary surveillance are particularly important because aspiration pneumonia is not caused by swallowing impairment alone. Oral microbial burden, impaired airway clearance, reduced consciousness, immobility, and respiratory vulnerability all influence whether aspiration develops into infection. Best-practice recommendations therefore include oral care as part of dysphagia management after stroke, and stroke-specific guidance explicitly links swallowing, nutrition, and oral care within the same clinical pathway (1, 23, 28). Early monitoring for signs and symptoms of aspiration should therefore be part of routine acute PSD care rather than a delayed response after pneumonia is suspected. These early supportive decisions create the conditions for safer rehabilitation and reduce the risk that dysphagia-related complications interrupt recovery. The acute-phase screening and instrumental assessment pathway is summarised in Table 1.

**Table 1:** Acute-phase screening and assessment pathway.

Clinical step	Who and when	Main question	Action or escalation	Evidence
Screen before oral intake	All patients with acute stroke; before food, fluid, or oral medication	Is unsupervised oral intake potentially unsafe?	If failed or uncertain: restrict unsafe intake; set hydration, nutrition, and medication routes; refer for CSE	(1, 4, 21, 24)
Bedside triage	Trained staff using a validated local tool	What immediate pathway is needed?	A screen identifies risk; it does not diagnose physiology or exclude silent aspiration	(1, 4, 24, 29)
Clinical swallowing examination	Failed screen, clinical predictors, or persistent concern	Safety, secretion control, likely phenotype, and immediate supports	Set interim diet/fluid, supervision, positioning, medication plan, and indication for FEES/VFSS	(1, 4, 29)
FEES	High risk, bedside uncertainty, secretion concern, fatigue, or limited transport tolerance	Secretions, residue, penetration/aspiration, sensation, and airway protection	Use findings to guide intake, airway protection, rehabilitation targets, and reassessment	(1, 23, 25, 29)
VFSS	Need for oral/pharyngeal biomechanics, bolus-flow analysis, or strategy testing	Which physiology and compensatory manoeuvres explain or improve performance?	Use findings to guide texture, posture, manoeuvres, feeding route, and rehabilitation targets	(1, 26, 29)

CSE, clinical swallowing examination; FEES, flexible endoscopic evaluation of swallowing; VFSS, videofluoroscopic swallowing study.

For consistency throughout this Review, overt signs and symptoms of aspiration (hereafter ‘aspiration warning signs’) include coughing or choking during or after intake, a wet or gurgly voice, new breathlessness or increased work of breathing, oxygen desaturation, fever, recurrent chest infection, and otherwise unexplained respiratory or functional deterioration. Silent aspiration may occur without these signs; therefore, absence of cough does not exclude aspiration, and instrumental assessment is required when clinical concern persists. Subsequent sections and tables refer back to the aspiration warning signs defined here (1, 2, 25, 29).

## 4 Rehabilitation across acute and subacute care: standard therapy and phenotype-based intensification modules

### 4.1 Standard swallowing rehabilitation as the platform

Once medically stable, during acute rehabilitation and the subacute phase, care should be built on standard swallowing therapy rather than isolated device-based or technique-specific interventions. Standard therapy directly addresses swallowing safety, bolus control, airway protection, oral intake progression, and functional eating, and usually includes compensatory strategies, postural adjustment, bolus and texture modification, swallowing manoeuvres, task-specific practice, oropharyngeal strengthening, sensory stimulation, and caregiver education (1, 4).

Adjunctive interventions are most meaningful when they intensify or support a coherent rehabilitation programme. Neuromuscular electrical stimulation, non-invasive brain stimulation, respiratory muscle training, and biofeedback have shown potential benefits in selected patients with PSD, but most evidence interprets them as additions to standard swallowing therapy rather than replacements (21, 30, 31). Effective management commonly combines compensatory, rehabilitative, and adjunctive approaches, with treatment selection depending on physiological impairment, severity, and patient tolerance (30).

Thus, the clinical question is not whether one technique is universally superior, but how standard therapy can be individualised and intensified. Patients with poor airway protection may need compensatory strategies and aspiration-risk control before active strengthening; those with reduced hyolaryngeal excursion may benefit from targeted exercise; and those with poor endurance or respiratory vulnerability may require respiratory training or pacing strategies alongside swallowing practice (1, 21). Standard swallowing rehabilitation is therefore the organising platform on which phenotype-based intensification modules should be selected, sequenced, and evaluated.

### 4.2 Definition and selection of phenotype-based intensification modules

For this Review, a phenotype-based intensification module is an optional, time-limited adjunct added to standard swallowing rehabilitation to address a defined physiological or functional target. The umbrella includes targeted exercise modules (for example, CTAR or lingual resistance), NMES, rTMS or tDCS, RMT, and biofeedback. It is not a separate treatment tier or a device list; selection also incorporates patient values and preferences, contraindications and safety, available resources and support, and observed response and tolerance. These modules are not part of the initial hyperacute safety gate. They may begin during acute rehabilitation once medical stability and participation permit and continue through subacute care, with modification or withdrawal according to response.

Selection begins with the dominant impairment and the patient’s stroke stage, treatment tolerance, and intended outcome. Current guidelines support early screening, structured assessment, and individualised management, but do not justify a fixed rehabilitation protocol for all patients with PSD (1, 4). This is clinically important because PSD may reflect different combinations of impaired airway protection, delayed swallowing initiation, reduced hyolaryngeal excursion, poor bolus propulsion, pharyngeal residue, respiratory vulnerability, cognitive impairment, and reduced endurance (15–19).

In the hyperacute and early acute stage, patients with unstable consciousness, poor secretion control, severe aspiration risk, or medical instability may be better served by safety-oriented management, compensatory strategies, nutrition and hydration planning, and reassessment before active intensification (1, 11). In the subacute stage, when patients can participate reliably, active modules can be matched to dominant impairments: CTAR or related strengthening for reduced hyolaryngeal excursion or suprahyoid weakness; lingual resistance or task-specific practice for reduced tongue pressure or bolus propulsion; repeated functional swallowing practice with feedback for impaired coordination (30–32).

Treatment goals should also shape selection. If the immediate goal is safe initiation of oral intake, compensatory strategies, texture modification, secretion management, and instrumental reassessment may be more relevant than aggressive strengthening. If the goal is swallowing efficiency, exercise-based therapy and selected intensification modules may be appropriate. If the goal is pneumonia prevention, oral hygiene, airway clearance, respiratory training, feeding supervision, and nutritional planning should be integrated with swallowing therapy (1, 11, 21). This phenotype- and stage-based approach is more useful for stroke clinicians than a simple hierarchy of interventions because it links treatment choice to physiological deficit, risk profile, participation capacity, and clinically meaningful outcomes. These goal-directed choices operationalise the phenotype-based intensification module defined above.

### 4.3 Exercise-based intensification modules

Exercise-based rehabilitation should be prescribed according to swallowing physiology rather than as a uniform package. It is usually combined with compensatory strategies, texture modification, and task-specific swallowing practice because patients often need both immediate safety support and longer-term functional restoration (1, 4, 30). Postural strategies such as chin tuck, head turn, or upright positioning may reduce aspiration risk in selected patients, but should be guided by observed swallowing physiology rather than applied universally (1, 30).

Task-specific practice and manoeuvre-based training, including effortful swallow, Mendelsohn manoeuvre, supraglottic swallow, and other targeted exercises, may be selected for deficits such as reduced tongue base retraction, impaired pharyngeal pressure generation, delayed swallow initiation, or reduced hyolaryngeal excursion (4, 30, 32). However, swallowing exercise dosage varies substantially across studies, underscoring the need for clearer reporting and individualised progression (32). CTAR is a better studied strengthening approach for suprahyoid activation and hyolaryngeal excursion, although trial protocols and outcomes remain heterogeneous (31). Broader reviews also suggest that CTAR, Shaker-type exercise, tongue-pressure resistance training, and related approaches may improve swallowing outcomes, with effects modified by stage, severity, adherence, and co-interventions (33).

Clinically, patients with poor airway protection may first require compensatory strategies and close monitoring; those with reduced hyolaryngeal excursion may be candidates for CTAR or related suprahyoid-focused exercises; those with reduced tongue strength or bolus propulsion may benefit from lingual resistance training; and those with poor coordination may require repeated task-specific practice with feedback (1, 4, 30, 31). Progression should be reviewed against predefined functional goals and observed response.

### 4.4 Adjunctive neurostimulation and device-based modules

The evidence base for adjunctive neurostimulation and device-based therapies remains heterogeneous. NMES may support peripheral muscle activation or sensory facilitation, and meta-analytic evidence suggests benefit when combined with conventional swallowing therapy (22). However, interpretation remains conditional because protocols vary in electrode placement, stimulation parameters, dose, severity, comparators, and outcomes (33).

Non-invasive brain stimulation is biologically plausible because PSD recovery involves reorganisation of bilateral swallowing networks. Evidence suggests potential benefit for rTMS and tDCS, with rTMS appearing favourable in some network analyses, but stimulation site, hemisphere selection, timing, frequency, session number, and responder profiles remain insufficiently standardised (34–36). Respiratory muscle training may be more relevant for patients with weak cough, reduced respiratory reserve, impaired airway protection, or poor respiratory-swallow coordination (21, 37). Biofeedback may be useful when motor learning, coordination, engagement, or adherence requires augmented visual or auditory feedback (38).

Overall, these approaches should be selected only when they address a plausible therapeutic target: NMES for peripheral activation or sensory facilitation, rTMS/tDCS for cortical network modulation in specialist or research-informed settings, RMT for airway protection and respiratory-swallow coordination, and biofeedback for motor learning and adherence (21, 38). When used, the target outcome, dose, concurrent standard therapy, clinical response, and adverse effects should be documented.

## 5 Supportive and goal-concordant care: nutrition, feeding, oral care, pneumonia prevention, and palliative care

### 5.1 Nutrition as an enabling condition for rehabilitation

Nutrition should be regarded as an enabling condition for PSD rehabilitation rather than an adjunctive issue after swallowing therapy. Stroke is often accompanied by dysphagia, reduced consciousness, fatigue, dependency, and reduced oral intake, all of which may compromise nutritional and hydration status (11). Clinical nutrition guidance states that dysphagia, aspiration pneumonia, and insufficient nutritional intake contribute to worse outcomes after stroke, supporting integration of nutrition into early and continuing dysphagia management (11).

The relationship between dysphagia and malnutrition is bidirectional: dysphagia limits safe intake, while malnutrition may reduce muscle strength, immune function, wound healing, training tolerance, and rehabilitation resilience (11, 20, 39). A systematic review found increased odds of malnutrition in patients with post-stroke dysphagia, supporting combined assessment of swallowing and nutritional status (40). Nutrition management should therefore include nutritional risk screening, dietary management, enteral nutrition when needed, tube-feeding care, education, and follow-up (27).

Clinically, rehabilitation intensity cannot be planned without considering nutritional reserve. Patients who are nil by mouth, on poorly tolerated texture-modified diets, or taking inadequate fluids may be unable to sustain swallowing exercises, respiratory training, or broader stroke rehabilitation. Dehydration and malnutrition are common after stroke and are associated with poorer outcomes (20). Nutritional screening, hydration planning, dietetic review, and timely oral versus enteral feeding decisions should therefore occur alongside swallowing assessment and therapy (11, 27).

Nutritional adequacy should be assessed through a two-step process. A validated screening tool should be used within 24–48 h of admission and repeated during the admission, at transitions of care, and whenever intake or clinical status changes. The Malnutrition Universal Screening Tool (MUST) or Nutritional Risk Screening 2002 (NRS-2002) may be used in acute care according to local validation, while the Mini Nutritional Assessment–Short Form (MNA-SF) is a reasonable option for older adults in rehabilitation or community settings (11, 20, 21, 27, 39).

A positive screen should trigger dietitian-led assessment of recent weight and body-mass-index trajectory, muscle mass or strength, actual food and fluid intake over 24–72 h relative to estimated requirements, feeding assistance, meal duration and fatigue, acceptance of texture-modified diets, hydration and fluid balance, tube tolerance and gastrointestinal symptoms, and inflammation or disease burden. Under the Global Leadership Initiative on Malnutrition (GLIM) framework, diagnosis requires at least one phenotypic criterion and one aetiologic criterion; serum albumin or prealbumin should not be used alone to diagnose inadequate nutrition (39, 41).

### 5.2 Route-of-feeding decisions and reassessment

Feeding-route decisions should be dynamic rather than one-off choices between oral and non-oral feeding. Texture-modified diets and thickened liquids may reduce immediate aspiration risk in selected patients, but should be prescribed after appropriate swallowing assessment and reassessed as swallowing physiology, alertness, endurance, hydration, and preference change (1). Prolonged or overly restrictive diets may reduce fluid intake, eating pleasure, adherence, and social participation if not reviewed regularly (12, 13).

For insufficient oral intake, early enteral feeding should maintain nutrition and hydration while recovery is monitored. The ESO/ESSD guideline suggests nasogastric enteral nutrition for PSD with insufficient oral intake. Clinical nutrition guidance recommends tube feeding when severe dysphagia is expected to last more than several days (1, 11). Nasogastric feeding should be viewed as a supportive bridge, not rehabilitation failure. Feeding route should be reassessed because swallowing status, tube tolerance, and nutritional goals may change (11, 27).

Percutaneous endoscopic gastrostomy (PEG) may be appropriate when prolonged enteral feeding is expected, but it should sit within a reassessment pathway rather than mark an irreversible endpoint. Canadian Stroke Best Practices recommends PEG when prolonged enteral nutrition is required, for example 4 weeks or longer (21). Here, the ‘very early acute phase’ refers to the first 7 days after stroke. Nasogastric feeding is preferred initially; when enteral feeding is expected for more than 28 days, PEG is generally considered once clinically stable, commonly 14–28 days after stroke. Earlier gastrostomy may be justified for prolonged ventilation or repeated nasogastric intolerance or removal after multidisciplinary review (27).

When gastrostomy is indicated, the access route should be individualised. In an entropy-balanced retrospective analysis of 217 adults with dysphagia after cerebral infarction, PEG and radiologically inserted gastrostomy (RIG) achieved similar times to goal feeding, while RIG was associated with lower odds of selected perioperative events. Because the evidence was observational and may reflect local referral patterns, it does not establish universal superiority; comorbidity, anatomy, local expertise, anticipated duration, and patient preferences should guide selection (42).

Each feeding decision should specify the current plan, reassessment timing, responsible professional, criteria for diet advancement or restriction, and triggers for earlier review, including new or worsening aspiration warning signs (Section 3.4), reduced intake, weight loss, dehydration, or tube intolerance (1, 11, 12). In a full-cycle model, feeding-route decisions should remain provisional, goal-oriented, and revisable.

### 5.3 Oral care and aspiration pneumonia prevention

Aspiration pneumonia prevention should not be reduced to aspiration detection alone. Pneumonia develops through interactions among impaired swallowing safety, oral microbial burden, reduced cough effectiveness, impaired consciousness, immobility, respiratory reserve, feeding dependence, and nursing quality (1, 2, 43). Meta-analytic evidence shows that post-stroke dysphagia is associated with substantially higher pneumonia risk (2).

Oral hygiene is central because aspirated material becomes more dangerous when it contains pathogenic oral bacteria. Poor oral hygiene is associated with respiratory tract infections and is recognised as an aspiration-pneumonia risk factor after stroke (44). Stroke-specific best-practice recommendations place swallowing, nutrition, and oral care within the same pathway, including oral care protocols, positioning, and feeding support to reduce choking and aspiration pneumonia risk (21).

Clinical studies support this integrated approach. Early systematic dysphagia screening combined with intensified oral hygiene reduced X-ray verified pneumonia after stroke (45). Intensified oral hygiene care has also been evaluated as part of stroke-associated pneumonia prevention, although effects may vary by setting and baseline risk (46). Consensus work continues to emphasise oral care, respiratory infection prevention, hydration, and nutrition as interconnected components of dysphagia management (28).

Cough effectiveness and pulmonary surveillance should be addressed alongside oral hygiene. Patients with weak cough, reduced alertness, poor secretion clearance, tube feeding, or limited mobility may need closer monitoring for the aspiration warning signs defined in Section 3.4, together with secretion clearance, feeding tolerance, and delirium (1, 47). A practical PSD pathway should specify oral assessment, cleaning frequency, denture and residue management, antiseptic use when indicated, and when oral-health professionals should be involved (43). By integrating oral hygiene, cough support, feeding assistance, positioning, and pulmonary surveillance, clinicians can address how dysphagia becomes pneumonia rather than focusing only on aspiration during a single swallow.

### 5.4 Palliative care, shared decision-making, and comfort-focused management

Palliative care may be integrated at any stage of the stroke pathway and should not be restricted to the last days of life. Early consultation is particularly relevant after severe stroke, with persistent high-burden dysphagia, uncertain prognosis, recurrent aspiration-related illness, or preference-sensitive decisions about clinically assisted nutrition and hydration. Its role is to improve prognostic communication, clarify goals and acceptable trade-offs, and support the patient, family, and multidisciplinary team (21, 48).

Shared decisions should address the expected benefits and burdens of nasogastric feeding, PEG or RIG, comfort-focused or acknowledged-risk oral intake, secretion management, thirst, dyspnoea, caregiver burden, and advance-care planning. Dysphagia clinicians and dietitians remain involved when goals shift toward comfort, adapting texture, positioning, mouth care, and feeding assistance to minimise distress. Decisions, responsible clinicians, review triggers, and the patient’s values should be documented and revisited as prognosis or swallowing changes. A trigger-based interdisciplinary quality-improvement project increased goals-of-care discussions before gastrostomy in patients with stroke and feeding problems, but comparative outcome evidence remains limited (48).

## 6 Beyond discharge: transitional care, community follow-up, and implementation barriers

### 6.1 The post-discharge gap

The weakest part of current PSD management is often the period after discharge. Acute stroke pathways increasingly emphasise early screening, risk identification, instrumental assessment, nutrition planning, and aspiration-pneumonia prevention, but post-discharge care is much less consistently specified (1, 4). A recent scoping review of long-term PSD guidelines found that recommendations for follow-up care are sparse and lack detailed guidance; only a minority of guidelines provided specific follow-up recommendations, and guidance on reassessment intervals, referral pathways, nutritional goals, oral care, and community-based support remained limited (5).

This gap has practical consequences. Patients may leave hospital with broad instructions such as “continue swallowing exercises,” “follow a modified diet,” or “avoid aspiration,” but without a clearly defined reassessment date, responsible clinician, escalation rule, or pathway for diet advancement. Canadian Stroke Best Practices recommends that people with stroke, their families, and caregivers should receive interdisciplinary education on swallowing, aspiration prevention, and feeding recommendations, but such recommendations still require local implementation through explicit discharge and follow-up procedures (21). Longitudinal qualitative evidence similarly shows that patients with PSD and family caregivers experience multiple transitions from admission to discharge home, with dynamic needs across hospitalisation, rehabilitation transfer, and home care (12).

The post-discharge gap is not merely an organisational inconvenience; it may affect recovery. In the STROKE CARD trial, intensified post-stroke care improved long-term dysphagia recovery within 1 year after acute ischaemic stroke, suggesting that targeted follow-up and structured care may influence swallowing outcomes rather than simply monitor them (13). Emerging evidence also suggests that evidence-based discharge planning may improve discharge readiness, self-management, safe feeding, and reduce aspiration or unplanned readmission among stroke patients with dysphagia, although further validation in broader settings is still needed (49). These findings support the need to define PSD care beyond the hospital episode.

Therefore, discharge should not be treated as the endpoint of PSD management. It should be a structured transition point at which the swallowing status, aspiration risk, diet and fluid recommendations, feeding route, oral care plan, caregiver instructions, warning signs, reassessment timing, and referral route are explicitly documented. Without these elements, the gains achieved during inpatient screening, rehabilitation, nutrition support, and oral care may not translate into sustained recovery at home or in the community.

### 6.2 What discharge planning should include

Discharge planning for patients with PSD should translate inpatient assessment into an executable home- or community-based care plan. At minimum, it should document the patient’s current swallowing status, aspiration risk, diet and fluid recommendations, feeding route, oral care requirements, warning signs, next reassessment plan, and the responsible service or team. This is consistent with stroke best-practice guidance stating that patients, families, and caregivers should receive interdisciplinary education on swallowing, aspiration prevention, and feeding recommendations, and that nutrition and hydration status should be screened repeatedly during admission and after discharge to the community (21).

The first element is a clear summary of current swallowing status and aspiration risk. Discharge documents should specify whether the patient has passed bedside screening, undergone clinical swallowing examination, received FEES or VFSS, and whether aspiration, penetration, residue, poor secretion control, or fatigue-related deterioration has been observed (1). This information is essential because community clinicians and caregivers cannot safely update diet texture, fluid consistency, medication route, or feeding assistance without knowing the physiological basis of the inpatient recommendation.

The second element is a precise diet, fluid, and feeding-route plan. Instead of broad advice such as “soft diet” or “swallowing precautions,” discharge instructions should specify food texture, fluid consistency, supervision level, feeding posture, pace, bolus size, use of compensatory manoeuvres, medication administration route, and whether oral intake, nasogastric feeding, gastrostomy feeding, or mixed feeding is currently recommended (1, 11, 21). These details should be paired with nutrition and hydration goals, because discharge on a modified diet without monitoring intake may increase the risk of dehydration, malnutrition, and poor rehabilitation tolerance (11, 27).

The third element is caregiver-facing instruction. Families and caregivers should be taught how to position the patient, assist feeding, maintain oral hygiene, recognise aspiration warning signs (Section 3.4) and nutritional concerns such as reduced intake, weight loss, dehydration, or increasing fatigue (21, 44). Longitudinal qualitative evidence shows that patients with PSD and their caregivers experience changing needs across hospitalisation, rehabilitation transfer, and return home, supporting the need for discharge education that is concrete, repeated, and adapted to the home context (12).

The fourth element is a scheduled reassessment and escalation pathway. Discharge planning should state when swallowing will be reassessed, who is responsible, and what findings should trigger earlier review or urgent referral. A scoping review of long-term PSD guidance found that follow-up recommendations are often sparse and insufficiently detailed, particularly regarding reassessment timing, referral pathways, nutritional goals, oral care, and community support (5). Emerging clinical evidence suggests that structured discharge planning can improve discharge readiness, self-management, safe feeding, and reduce aspiration or unplanned readmission among stroke patients with dysphagia, further supporting the need for explicit post-discharge pathways (49). Therefore, an adequate discharge plan should function not as a static instruction sheet, but as a care-transfer document that connects the inpatient stroke team, rehabilitation professionals, primary care, community services, dietitians, speech-language therapists, oral-care providers, caregivers, and the patient. Minimum discharge and follow-up elements for PSD continuity of care are listed in Table 2.

**Table 2:** Minimum discharge and follow-up elements for continuity of post-stroke dysphagia care.

Element	Required discharge content	Owner	Earlier-review trigger	Evidence
Swallowing status	CSE/FEES/VFSS findings; aspiration/penetration, residue, secretion control, fatigue; current uncertainty	Stroke team and SLT	New or worsening aspiration warning signs (Section 3.4) or reduced alertness	(1, 4, 23, 25, 29)
Diet, fluid, feeding, medication	Texture and fluid level; supervision; oral/NG/PEG/RIG/mixed route; medication route; nutrition/hydration targets	Physician, SLT, dietitian, nurse, pharmacist	Reduced intake, dehydration, weight loss, tube intolerance, or medication omission	(1, 11, 21, 27, 42)
Oral care and pneumonia prevention	Cleaning frequency; denture/residue care; positioning; feeding assistance; secretion clearance and pulmonary monitoring	Nurse, caregiver, SLT, oral-health team	Aspiration warning signs, oral infection, or secretion deterioration	(1, 2, 28, 43–47)
Warning signs and escalation	Teach the Section 3.4 aspiration warning signs; specify who to contact, urgency, and backup route	Named hospital/community service	Any new or worsening warning sign or otherwise unexplained decline	(1, 2, 12, 47, 49)
Reassessment	Date, mode, clinician, criteria for diet advancement/restriction, and need for FEES/VFSS	Named service; primary care informed	Missed review, clinical change, or uncertain diet progression	(5, 12, 13, 49, 63)
Caregiver, community, digital support	Teach-back and demonstrated competency; appointment before discharge; closed-loop referral; accessible hybrid support	SLT, nurse, rehabilitation/community team, coordinator	Caregiver uncertainty or overload, access barrier, non-completed referral, or poor adherence	(12, 20, 50, 52–61)
Goals and palliative input	Patient values; expected benefits/burdens of feeding routes; comfort measures; review triggers; advance-care plan	Stroke team, palliative care, SLT, dietitian, patient/family	Preference-sensitive conflict, high symptom burden, recurrent illness, or prognostic change	(21, 48)

CSE, clinical swallowing examination; FEES, flexible endoscopic evaluation of swallowing; NG, nasogastric; PEG, percutaneous endoscopic gastrostomy; PSD, post-stroke dysphagia; RIG, radiologically inserted gastrostomy; SLT, speech-language therapist; VFSS, videofluoroscopic swallowing study.

### 6.3 Long-term outcomes and caregiver burden

Long-term PSD should not be understood only as persistent impairment in swallowing physiology. For many stroke survivors, dysphagia becomes a chronic condition that affects daily routines, eating confidence, social participation, family roles, and quality of life. Qualitative evidence from individuals living with persisting post-stroke dysphagia shows that swallowing difficulties can create ongoing restrictions in everyday life, including uncertainty around eating, dependence on others, altered meal experiences, and the need for continued dysphagia management beyond the initial hospital episode (50). Quantitative studies also show that dysphagia has a detrimental effect on quality of life in stroke patients, particularly in psychosocial domains and social isolation (51).

Eating is a social and emotional activity, not simply a nutritional task. Patients with PSD may avoid shared meals, feel embarrassed by coughing or modified diets, fear choking or aspiration, and experience loss of autonomy when eating requires supervision or tube feeding. A longitudinal qualitative study of patients with PSD and family caregivers found that their experiences changed dynamically across admission, transfer to rehabilitation institutions, and return home, with substantial challenges in each transition phase and a need for professional collaboration, follow-up support, and community or social resources (12). Another interview study of stroke survivors and informal caregivers also highlighted that dysphagia-related experiences include fear, uncertainty, disrupted routines, and the need for clearer information and support during recovery (52).

Caregiver burden is a parallel and often under-recognised outcome. Family carers may need to prepare texture-modified meals, supervise feeding, monitor aspiration signs, provide oral care, manage tube feeding, encourage exercises, and decide when to seek medical help. Studies focusing on caregivers of stroke survivors with dysphagia show that informal care can involve substantial burden, and family carers’ experiences include anxiety around safe swallowing, responsibility for food and fluid modification, and concern about choking or respiratory complications (53, 54). These findings indicate that PSD follow-up should evaluate not only swallowing scales and diet level, but also caregiver capacity, confidence, training needs, and emotional strain.

Long-term outcome assessment should therefore extend beyond whether a patient resumes oral intake. A patient may eat orally yet still have dietary restriction, fear of eating, reduced enjoyment, avoidant social behaviour, inadequate hydration, or recurrent respiratory events. Emerging evidence also suggests that dysphagia-related quality of life is linked with nutritional status and psychological factors after stroke, reinforcing the need to integrate physical, nutritional, and psychosocial follow-up (55). In a full-cycle model, long-term PSD care should include periodic reassessment of swallowing safety, nutrition and hydration, pneumonia risk, patient-reported quality of life, social participation, and caregiver burden, rather than ending follow-up once a basic oral diet is achieved.

### 6.4 Community and digitally supported follow-up

Community and digitally supported follow-up may help convert PSD care from a hospital-centred episode into a continuous rehabilitation pathway. After discharge, patients and caregivers often need support with diet texture, fluid consistency, oral care, feeding posture, swallowing exercises, tube-feeding management, warning-sign recognition, and decisions about when to seek reassessment (12). Virtual stroke rehabilitation recommendations state that technology-supported rehabilitation can be delivered at different stages of the care continuum, alone or in hybrid formats, and may improve access to rehabilitation providers, home monitoring, community reintegration, mental health, and activities of daily living (20, 56). Although PSD-specific digital evidence is still emerging, these principles are highly relevant to patients whose swallowing management must continue at home.

Remote reassessment is one potential application. Digital follow-up could be used to screen for changes in swallowing safety, oral intake, hydration, weight, respiratory symptoms, tube tolerance, adherence to oral care, and caregiver concerns. It should not be used as an uncritical substitute for FEES or VFSS when instrumental assessment is needed, but it may help identify patients who require earlier in-person review. A recent study on telemedicine for dysphagia rehabilitation in home-care patients suggests that remote care may help maintain swallowing-related management when direct home visits are limited, although the evidence remains preliminary and should be interpreted cautiously (57).

Home exercise feedback is another promising direction. Swallowing exercises are difficult for patients to perform correctly without supervision, and adherence may decline after discharge. Digital platforms could support video-guided exercises, symptom diaries, meal or fluid logs, reminders, caregiver coaching, and therapist feedback. Recent discussion of digital technology in swallowing rehabilitation highlights potential advantages such as personalised exercises, remote monitoring, real-time feedback, and improved continuity between clinicians and patients (58). Community-based rehabilitation evidence also suggests that swallowing training combined with health education can improve swallowing-related outcomes and quality of life in older stroke survivors living outside hospital settings (59).

Caregiver support should be a central function of digital follow-up, not a secondary feature. Family caregivers often carry responsibility for meal preparation, safe feeding, texture modification, oral care, symptom monitoring, and help-seeking decisions, while also experiencing uncertainty and emotional burden (54). Remote education, structured checklists, shared videos, and rapid access to professional advice may reduce avoidable uncertainty and help caregivers recognise the aspiration warning signs defined in Section 3.4 and changes in intake, hydration, weight, or alertness that require review (12).

Finally, digital tools could improve cross-setting information sharing. A practical PSD follow-up system should allow inpatient findings, FEES/VFSS results, diet and fluid prescriptions, oral care instructions, feeding-route decisions, exercise plans, reassessment dates, and responsible services to be visible across hospital, rehabilitation, primary care, community, and family settings. This is consistent with broader global stroke service recommendations that emphasise organised stroke systems, rehabilitation access, and continuity of care beyond the acute hospital. The next step is not simply to add a smartphone application to PSD care, but to embed digital tools into a defined hospital-home-community pathway with clear escalation rules, data governance, clinician responsibility, and outcomes that matter to patients and caregivers.

### 6.5 Implementation barriers in discharge and community care

PSD-specific implementation research remains limited, but qualitative dysphagia studies and broader stroke-transition evidence identify recurrent barriers: inconsistent or disconnected discharge education and documentation; absence of a named owner, reassessment date, or escalation route; limited access to speech-language therapy, dietetics, FEES, or VFSS; rural and under-resourced service gaps; transport, cost, mobility, language, health-literacy, and digital-access constraints; poor awareness of available resources and difficulty navigating them; caregiver workload and low confidence; and loss to follow-up after referral (12, 50, 52, 54, 60, 61).

Implementation responses should therefore include a standardised minimum transfer dataset, teach-back and hands-on verification of caregiver competency, a named service and appointment before discharge, closed-loop referral confirmation, risk-stratified follow-up, training for community teams, and hybrid digital support that does not exclude people who lack devices, connectivity, or digital literacy. These are practice-based pathway proposals and require evaluation across different health systems.

A post-stroke navigator or named care coordinator is a plausible mechanism for linking hospital, rehabilitation, primary care, community services, and families, but its PSD-specific effectiveness has not been established. Future work should co-design and test navigation and transition interventions with stroke survivors, caregivers, clinicians, community providers, and rural and urban stakeholders, while measuring access, referral completion, loss to follow-up, clinical outcomes, caregiver burden, and equity.

## 7 Discussion: evidence gaps and priorities for future care pathways

### 7.1 Evidence certainty and implications for practice

The current evidence base for PSD management is uneven. High-consensus practices include dysphagia screening before oral intake, timely escalation to clinical swallowing examination and FEES or VFSS in high-risk patients, and integration of nutrition, hydration, oral care, and pneumonia prevention into stroke pathways (21, 29). These practices are supported by evidence that dysphagia is associated with aspiration, pneumonia, malnutrition, dehydration, and mortality (2), and that oral microbial burden and care quality are clinically relevant to respiratory outcomes after stroke (45).

By contrast, several rehabilitation technologies remain promising but not definitive. NMES may improve swallowing function when combined with conventional therapy, but studies vary in electrode placement, stimulation parameters, treatment dose, comparators, and outcomes (22). Non-invasive brain stimulation, including rTMS and tDCS, is biologically plausible and supported by network meta-analytic evidence, but stimulation site, hemisphere selection, timing, frequency, and responder characteristics remain insufficiently standardised (62). Respiratory muscle training may be useful for patients with weak cough, poor airway protection, or reduced respiratory reserve, but available studies are limited by small samples, variable protocols, and incomplete long-term outcomes (37). Biofeedback may support motor learning and engagement, although current network evidence should be interpreted cautiously because of limited and heterogeneous trials (38). These modalities should therefore be considered conditional, phenotype-based intensification modules rather than routine substitutes for standard swallowing therapy, nutrition support, oral care, and reassessment.

Table 3 distinguishes guideline-supported statements from conditional evidence and practice-based or emerging proposals so that recommendations for discharge, digital health, community follow-up, and palliative care are not presented with greater certainty than the underlying evidence permits.

**Table 3:** Narrative evidence status and limits of inference across the full-cycle pathway.

Domain	Narrative evidence status	What the Review supports	Caveat or research need
Acute screening	High-consensus / guideline-supported	Screen before any oral intake or medication and link failure or uncertainty to a defined pathway	Tool performance and local implementation vary
Clinical and instrumental assessment	High-consensus / guideline-supported	Use CSE plus FEES or VFSS when risk, uncertainty, or a complex management decision persists	Modality choice depends on the clinical question, stability, expertise, and access
Nutrition, feeding, and oral care	Guideline-supported, with context-dependent decisions	Screen nutritional risk; quantify intake; support hydration; individualise enteral access; provide oral-care and pneumonia-prevention measures	Optimal tools, timing, and access route require patient- and system-level judgement
Standard swallowing rehabilitation	Guideline-supported platform; intervention effects vary	Combine compensatory and rehabilitative care around defined functional goals	Dose, progression, phenotype definitions, and long-term outcomes need better reporting
Exercise and adjunctive modules	Conditional / heterogeneous	Consider targeted exercise, NMES, rTMS/tDCS, RMT, or biofeedback as optional modules when a plausible target exists	Not universal prescriptions; protocols, comparators, outcomes, and responder profiles remain heterogeneous
Discharge planning and caregiver education	Consensus- and practice-supported; limited comparative evidence	Use a minimum transfer dataset, teach-back, a named owner, a scheduled review, and explicit escalation rules	Implementation effectiveness and resource requirements need comparative evaluation
Digital and community follow-up	Emerging / practice-based	Use hybrid support for monitoring, education, adherence, and referral linkage within a defined pathway	Do not substitute for indicated instrumental assessment; evaluate equity and digital exclusion
Palliative care and navigation	Practice-based / emerging, supported by shared-decision principles	Offer palliative input for high burden or preference-sensitive decisions; test named coordinator or navigator models	PSD-specific effectiveness, timing, acceptability, and equity outcomes remain uncertain

The three categories are narrative interpretive labels and do not represent a formal GRADE certainty assessment. CSE, clinical swallowing examination; FEES, flexible endoscopic evaluation of swallowing; NMES, neuromuscular electrical stimulation; RMT, respiratory muscle training; rTMS, repetitive transcranial magnetic stimulation; tDCS, transcranial direct current stimulation; VFSS, videofluoroscopic swallowing study.

### 7.2 Limitations of this review

This is a narrative rather than systematic review. The search was English-language, iterative, and purposive; Google Scholar ranking is dynamic, and raw database hit counts were not retained for a deduplicated selection denominator. We did not perform formal risk-of-bias assessment, GRADE certainty rating, or quantitative pooling. The Review also spans heterogeneous clinical questions, populations, interventions, care settings, and outcomes, so a single meta-analysis would not yield a clinically coherent summary effect. The three narrative evidence tiers improve interpretive transparency but do not replace formal certainty assessment. Finally, the proposed full-cycle pathway is an evidence-informed implementation framework that requires prospective evaluation.

### 7.3 Research and implementation priorities

Key practice gaps remain. First, the optimal timing and frequency of reassessment after the acute phase are unclear. Current long-term PSD guidance provides limited detail on reassessment intervals, referral rules, nutritional goals, oral care, and community support (5). Future studies should develop risk-stratified reassessment pathways based on early dysphagia severity, aspiration risk, feeding route, nutritional status, respiratory vulnerability, cognitive participation, and caregiver capacity. Severe PSD is associated with substantial mortality, pneumonia risk, prolonged tube feeding, and poorer long-term recovery, supporting a stratified rather than uniform follow-up model (63).

Second, future trials should move beyond short-term surrogate outcomes such as PAS, FOIS, or diet level. Core outcomes should include pneumonia, nutritional recovery, hydration status, readmission, mortality, patient-reported swallowing-related quality of life, social participation, and caregiver burden. This broader outcome framework is consistent with evidence linking PSD to pneumonia and mortality (2), nutrition and hydration compromise (11), and long-term psychosocial burden among patients and caregivers (12).

Third, the field needs pathway-level evidence rather than more isolated small device trials. Intensified post-stroke care may improve long-term dysphagia recovery, suggesting that organised follow-up can influence outcomes (13). Future trials should test integrated models combining screening, instrumental assessment, phenotype-based rehabilitation, nutrition support, oral care, discharge planning, caregiver education, community reassessment, and escalation rules. Digital tools should be evaluated only when embedded in such clinical pathways, where they support reassessment, education, monitoring, feedback, and cross-setting coordination rather than functioning as stand-alone technologies (58).

Implementation studies should quantify what happens at the discharge–community interface: whether patients and caregivers understand the plan; whether referrals are accepted and completed; how access, transport, geography, cost, language, health literacy, digital exclusion, and caregiver capacity affect uptake; and where loss to follow-up occurs. Stakeholder co-design should precede intervention trials, and navigator or coordinator models should be compared with usual care using clinical, implementation, caregiver, and equity outcomes (60, 61).

## 8 Conclusion

Post-stroke dysphagia should be understood as a cross-stage, cross-setting, and multidisciplinary stroke management problem rather than an isolated swallowing symptom. Current consensus is strongest for early screening before oral intake, timely escalation to clinical and instrumental assessment in high-risk patients, standard swallowing rehabilitation, and integration of nutrition, hydration, oral care, and aspiration-pneumonia prevention into routine stroke care. Palliative-care input and shared decision-making should be available when prognosis, symptom burden, or feeding choices are preference-sensitive.

The next priority is to extend this foundation beyond the inpatient episode. Future PSD care should place greater emphasis on discharge transition, risk-stratified reassessment, caregiver education, community follow-up, and digitally supported coordination across hospital, rehabilitation, primary care, and home settings. This shift is particularly important because long-term PSD guidance remains underdeveloped, especially regarding reassessment intervals, referral rules, nutritional goals, oral care, and community support. Effective implementation also requires explicit ownership, closed-loop referral, and strategies addressing access, transport, navigation, caregiver capacity, digital exclusion, and loss to follow-up.
